# Genomic landscape of circulating tumor DNA in HER2-low metastatic breast cancer

**DOI:** 10.1038/s41392-024-02047-0

**Published:** 2024-12-09

**Authors:** Zongbi Yi, Kaixiang Feng, Dan Lv, Yanfang Guan, Youcheng Shao, Fei Ma, Binghe Xu

**Affiliations:** 1 Department of Radiation and Medical Oncology, Hubei Key Laboratory of Tumor Biological Behaviors, Hubei Cancer Clinical Study Center, Zhongnan Hospital of Wuhan University, Wuhan, China; 2 Department of Breast and Thyroid Surgery, Hubei Key Laboratory of Tumor Biological Behaviors, Hubei Cancer Clinical Study Center, Zhongnan Hospital of Wuhan University, Wuhan, China; 3 Department of Medical Oncology, Cancer Hospital of China Medical University, Liaoning Cancer Hospital, Shenyang, China; 4 Geneplus-Beijing, Beijing, China; 5 Department of Pathology and Pathophysiology, TaiKang Medical School (School of Basic Medical Sciences), Wuhan University, Wuhan, China; 6 Department of Medical Oncology, National Cancer Center/National Clinical Research Center for Cancer/Cancer Hospital, Chinese Academy of Medical Sciences and Peking Union Medical College, Beijing, China

**Keywords:** Breast cancer, Cancer genomics, Tumour heterogeneity

## Abstract

The large population of HER2-low breast cancer patients necessitates further research to provide enhanced clinical guidance. In this study, we retrospectively analyzed 1071 metastatic breast cancer (MBC) patients and the circulating tumor DNA (ctDNA) to investigate clinicopathological and genetic alterations of HER2-low MBC patients. The effect of HER2-low status on different treatment modalities was explored in two prospective clinical trials (NCT03412383, NCT01917279) and a retrospective study. Our findings suggest *TP53*, *PIK3CA*, and *ESR1* are frequently mutated genes in HER2-low MBC. Compared to the HER2-0 group, mutations observed in the HER2-low group are more closely associated with metabolic pathway alterations. Additionally, among patients with *ERBB2* mutations and treated with pyrotinib, the HER2-low group may experience superior prognosis when compared to the HER2-0 group. Notably, we did not find any statistically significant disparity in the response to chemotherapy, endocrine therapy, or CDK4/6 inhibitor therapy between HER2-0 and HER2-low breast cancer patients. Interestingly, within the subgroup of individuals with metabolic pathway-related gene mutations, we found that HER2-low group exhibited a more favorable response to these treatments compared to HER2-0 group. Additionally, dynamic analysis showed the HER2-low MBC patients whose molecular tumor burden index decreased or achieved early clearance of ctDNA after the initial two treatment cycles, exhibited prolonged survival. Moreover, we classified HER2-low MBC into three clusters, providing a reference for subsequent treatment with enhanced precision. Our study offers valuable insights into the biology of HER2-low MBC and may provide reference for personalized treatment strategies.

## Introduction

The prevalence of human epidermal growth factor receptor 2 (HER2)-positive breast cancer is ~20%–30% among all breast cancer cases, and it is linked to an unfavorable prognosis.^1,2^ The increased expression of the HER2 protein plays a vital role as an essential indicator for identifying patients who could potentially derive advantages from trastuzumab or alternative treatments that specifically target HER2.^3^ Currently, the classification of HER2-positive breast cancer is based on immunohistochemical staining (IHC) with a score of 3+ and/or amplified in situ hybridization (ISH).^4^ HER2-low (HER2 IHC 1+ or 2+/ISH−) breast cancer is excluded from HER2-targeted therapy, account for ~40%–60% of HER2-negative breast cancer.^5^ However, emerging clinical evidence indicates that the HER2-low breast cancer patients may potentially benefit from novel antibody–drug conjugates (ADC) that target the HER2, such as trastuzumab deruxtecan (T-DXd) and trastuzumab duocarmazine.^6–8^

The prognostic implications of HER2-low breast cancer remain inconclusive in comparison to HER2-0 (HER2 IHC 0) breast cancer across previous studies.^9,10^ A retrospective study revealed comparable outcomes in terms of overall survival (OS) and progression-free survival (PFS) between HER2-0 and HER2-low advanced breast cancer patients who administered eribulin or capecitabine, yielding no significant difference between the two groups.^11^ In addition, a multicenter, real-world data analysis found no significant difference in PFS between HER2-0 and HER2-low breast cancer patients treated with CDK4/6 inhibitors, suggesting that the response to CDK4/6 inhibitors is not significantly influenced by HER2 status.^12^ HER2-low breast cancer exhibits a higher incidence in hormone receptor (HR)-positive breast cancer as opposed to HR-negative breast cancer.^13^ Differences in the HR status could potentially introduce confounding variables that impact the prognosis of HER2-low tumors.^14^ In a study involving 5235 cases of HER2-negative breast cancer, a significant association was found between the occurrence of HER2-low and the presence of estrogen receptor (ER) expression. Adjusting for ER status, no prognostic difference between HER2-0 and HER2-low tumors.^15^ Conversely, some studies propose that the HER2-low status exerts an impact on patient outcomes. The study conducted by Guven et al. showed that HER2-low metastatic breast cancer (MBC) patients exhibited significantly higher rates of disease progression or mortality in HR-positive patients compared to those with HER2-0 MBC.^16^ However, Denkert et al. conducted a pooled analysis of 2310 patients with HER2-negative breast cancer and observed that among HR-negative tumor subtypes, patients diagnosed with HER2-low breast cancer exhibited significantly improved disease-free survival (DFS) and OS compared to those with HER2-0.^17^ The study conducted by Shi et al. indicated that there was no statistically significant difference in the complete response (CR) rate between the HER2-0 and HER2-low group among triple-negative breast cancer (TNBC) patients receiving neoadjuvant chemotherapy, while the 5-year OS of the HER2-low group was significantly longer than that of the HER2-0 group.^18^ Our previous analysis of the PALOMA-2 and PALOMA-3 trials suggests that patients with HER2-0 breast cancer may derive limited benefit from first-line treatment with CDK4/6 inhibitors. However, the combination of CDK4/6 inhibitor and endocrine therapy significantly enhances the prognosis of patients with HER2-low breast cancer.^19^ These findings suggest that further investigation is warranted to explore the prognostic implications of HER2-low status and the intrinsic molecular characteristics of HER2-low breast cancer.

Relevant clinicopathological and molecular features of HER2-low breast cancer may differ from those observed in HER2-0 breast cancer.^17,20^ Zhang et al. discovered that HER2-0 breast cancer exhibited a higher frequency of somatic mutations in p53 signaling, Fanconi anemia, checkpoint factors, and cell cycle pathways compared to HER2-low breast cancer.^21^ The study conducted by Schettini et al. discovered that HER2-low breast cancer exhibited decreased expression levels of genes associated with cell proliferation and increased expression levels of genes related to luminal characteristics when compared to HER2-0 tumors in the HR-positive group.^13^ The results of a study indicated that among patients with HR-positive breast cancer, those in the HER2-low subgroup exhibited a higher prevalence of *BRCA2* germline mutations compared to individuals in other HER2 status.^22^ However, due to the considerable heterogeneity observed in HER2-low breast cancer and the absence of a standardized HER2 detection platform, there is currently inadequate evidence to support categorizing HER2-low breast cancer as a distinct biological subtype. Additionally, it is noteworthy that the majority of previous studies on the molecular characteristics of HER2-low breast cancer primarily relied on primary tissue analysis. Given the limitations in sampling breast cancer, particularly metastatic cases, it is evidently imperative to investigate the circulating tumor DNA (ctDNA) features of HER2-low breast cancer. This will enable a wider range of breast cancer patients to benefit from the findings.

We undertook this study to investigate the clinical and molecular characteristics of HER2-low MBC in a substantial cohort consisting of 1071 patients who underwent ctDNA analysis, as well as in a prospective phase 2 clinical trial (NCT03412383), a prospective randomized phase 3 clinical trial (CAMELLIA study, NCT01917279) and a retrospective study. Furthermore, the prognosis of patients with HER2-low and HER2-0 breast cancer, who were treated with HER2 tyrosine kinase inhibitor (TKI), chemotherapy, endocrine therapy or CDK4/6 inhibitors therapy, and the prognostic differences in specific mutation characteristics between the two groups, were thoroughly discussed. Additionally, a preliminary exploration was conducted on the prognostic index of HER2-low and the clusters of HER2-low breast cancer.

## Results

### Cohort information and clinical characteristics

A total of 1071 MBC patients were enrolled in cohort 1 of this study, including 267 (24.9%) HER2-0 MBC patients, 488 (45.6%) HER2-low MBC patients, and 316 (29.5%) HER2-positive MBC patients, according to HER2 status by IHC and ISH. In addition, among HER2-low MBC patients, IHC 1+ patients accounted for 55% and IHC 2+/ISH− patients accounted for 45%. The median age at diagnosis for both HER2-low MBC patients and the overall cohort was 46 years (range: 24–82), excluding patients with unknown age. There were no notable variances observed in the age distribution when comparing HER2-low and HER2-0, as well as HER2-positive groups. In this cohort, 660 patients (61.6%) exhibited HR positivity, while the remaining 411 patients (38.4%) displayed HR negativity. A greater percentage of patients with HR-positive status was noted in the HER2-low group compared to those in the remaining two groups (73.6% vs 51.7%, 51.6%, *P* < 0.01), indicating a relatively higher prevalence of luminal breast cancer subtype in the HER2-low MBC. Correspondingly, there was a lower incidence of TNBC subtype in HER2-low MBC as compared to HER2-0 MBC (26.4% vs 48.3%, *P* < 0.01). Other baseline characteristics are shown in Table 1.

**Table 1:** Patient characteristics of cohort 1

Characteristics	No. of patients (%)				*P* value
	Total (*n* = 1071)	HER2-zero (*n* = 267)	HER2-low (*n* = 488)	HER2-positive (*n* = 316)	
Age					0.197
≤35	162 (15.1)	39 (14.6)	80 (16.4)	43 (13.6)	
35–60	757 (70.7)	192 (71.9)	352 (72.1)	213 (67.4)	
>60	114 (10.6)	30 (11.2)	45 (9.2)	39 (12.3)	
Unknown	38 (3.6)	6 (2.3)	11 (2.3)	21 (6.7)	
Histopathological					0.209
Ductal	972 (90.8)	235 (88.0)	450 (92.2)	287 (90.8)	
Lobular	43 (4.0)	15 (5.6)	18 (3.7)	10 (3.2)	
Mixed	2 (0.2)	0	2 (0.4)	0	
Other	54 (5.0)	17 (6.4)	18 (3.7)	19 (6.0)	
HER2 status					<0.001
IHC 0	267 (24.9)	267 (100.0)	0	0	
IHC 1+	268 (25.1)	0	268 (55.0)	0	
IHC 2+	269 (25.1)	0	220 (45.0)	49 (15.5)	
IHC 3+	267 (24.9)	0	0	267 (84.5)	
HR status					<0.001
Positive	660 (61.6)	138 (51.7)	359 (73.6)	163 (51.6)	
Negative	411 (38.4)	129 (48.3)	129 (26.4)	153 (48.4)	

*HER2* human epidermal growth factor receptor 2, *IHC* immunohistochemical staining, *HR* hormone receptor

### Somatic mutation landscapes of HER2-low MBC compared to HER2-0, and HER2-positive MBC

**Fig. 1:**
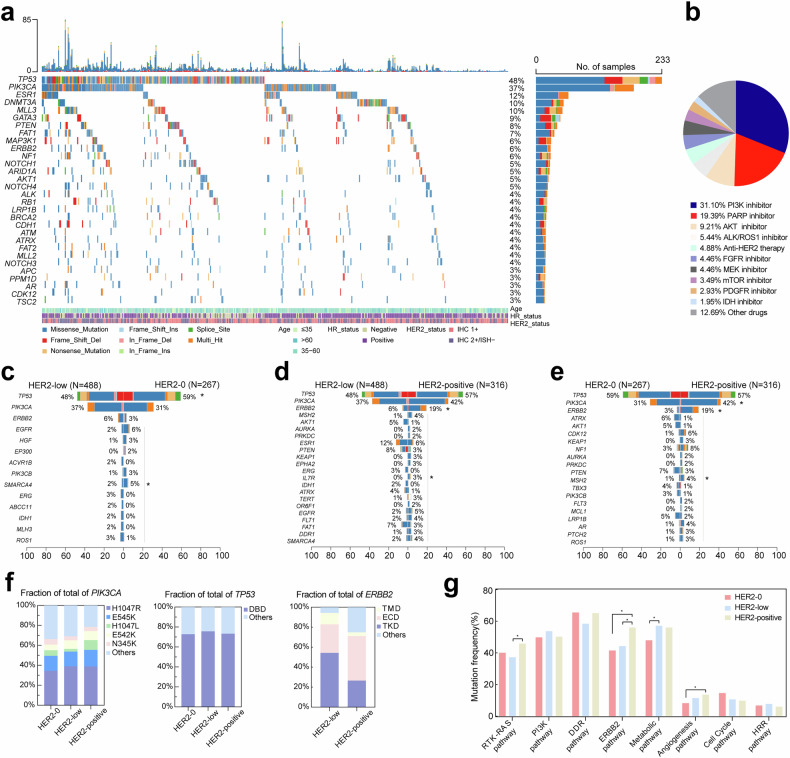
Mutation characteristics of MBC with different HER2 status. **a** The mutation profile of the top 30 commonly mutated genes in 488 patients with HER2-low MBC. **b** Percentage of actionable drugs approved by FDA. **c**–**e** Pairwise comparison of three groups of differentially mutated genes in HER2-low and HER2-0, HER2-positive. **f** Distribution of mutation hotspots of key genes (*PIK3CA*, *TP53*, *ERBB2*) in HER2-0, HER2-low and HER2-positive groups. **g** Characteristics analysis of oncogenic signaling pathways across the three groups. *P < 0.05

First, our study focused on the ctDNA genomic mutational alterations in patients diagnosed with HER2-low MBC. The predominant mutation type observed was missense mutation (77.4%), and the genes most frequently affected were *TP53* (47.8%), *PIK3CA* (37.1%), and *ESR1* (12.1%) (Fig. 1a). These genes were mutated at multiple sites (Supplementary Fig. 1a). Furthermore, the OncoKB database was utilized for annotating available molecular targeted therapies approved by the Food and Drug Administration (FDA),^23^ revealing that 181 patients (37.1%) could potentially benefit from PI3K inhibitors (such as alpelisib). Additionally, PARP inhibitors (such as olaparib) were found to be applicable in 88 patients (18.0%), while AKT inhibitors (such as capivasertib) were suitable for 60 patients (12.3%). Notably, a significant proportion of 327 patients (67.0%) exhibited actionable events ranging from 1 to 8 (Fig. 1b).

The mutant profiles of the HER2-low, HER2-0, and HER2-positive groups were subsequently compared. Supplementary Fig. 1b, c illustrates the mutation profiles of the HER2-0 and HER2-positive groups, while summarizing the differentially mutated genes across all three groups. In comparison to the HER2-0 group, a lower mutation frequency was observed for *TP53*, *EGFR*, and *HGF* in the HER2-low group, conversely, *IDH1*, *MLH3*, and *ROS1* exhibited higher mutation frequencies (Fig. 1c). When comparing with the HER2-positive group, a lower mutation frequency was found for *ERBB2* (which encode HER2), *MSH2*, and *TP53* in the HER2-low group, however, there was a higher mutation frequency for *ESR1* in this same group. Additionally, other genes such as *AKT1*, *PTEN*, and *FAT1* also displayed more frequent mutations (Fig. 1d). Notably significant differences were observed between *ERBB2* and *AKT1* mutation frequencies in both the HER2-0 and HER2-positive groups (Fig. 1e).

Furthermore, our study focused on comparing the distribution of hotspot mutations in *PIK3CA*, *TP53*, and *ERBB2* among the aforementioned three groups (Fig. 1f and Supplementary Fig. 1e–g). The predominant type of mutation observed in these genes within each group was missense mutation. Among *PIK3CA* mutations, H1047R was found to be predominant across all groups (34.6%, 39.0%, 38.9%, *P* = 0.722). In comparison to HER2-low MBC, HER2-positive MBC exhibited a higher frequency of mutations at the H1047L site (9.7% vs 2.7%, *P* = 0.004), while no significant difference was observed in other mutation sites. In addition, there was no significant difference in the frequency of DNA-binding domain (DBD) mutation in *TP53* among the three groups (72.8%, 75.6%, 73.3%, *P* = 0.775). Among the hotspot mutations in DBD, the HER2-positive group exhibited a higher prevalence of mutations at the R273 site compared to the other two groups (4.1%, 4.8%, 11.4%, *P* = 0.023). Given the low occurrence rate of *ERBB2* mutations in the HER2-0 group, our focus shifted towards assessing the mutation site profile of *ERBB2* in both HER2-low and HER2-positive groups, revealing a significantly higher mutation frequency within the tyrosine kinase domain (TKD) for HER2-low cases compared to HER2-positive cases (52.3% vs 26.6%, *P* < 0.001). Furthermore, it was observed that mutation sites within *ERBB2* were more dispersed in the HER2-positive group, with certain hotspot mutations such as p.V777L, p.D769Y/H, and p.L755S occurring more frequently within the HER2-low group.

We performed a comparative analysis of the classical oncogenic pathways of MBC across different HER2 status (Fig. 1g). Mutated genes within the ERBB2 and angiogenesis pathways exhibit dissimilarities between the HER2-0 and HER2-positive groups. The distinction between the HER2-positive group and the HER2-low group lies in a higher prevalence of mutations in genes within the RTK–RAS and ERBB2 pathways among the HER2-positive group. Notably, we found that the mutations observed in the HER2-low group are more closely associated with metabolic pathway alterations compared to those in the HER2-0 group.

Additionally, we also compared the blood tumor mutation burden (bTMB) of each group (Supplementary Fig. 1d). The median bTMB of HER2-low group was 3.64 (range: 0.91–77.27). The median bTMB of HER2-0 group was 4.55 (range: 0.91–100.91). While the median bTMB of HER2-positive group was 3.64 (range: 0.91–344.55). Overall, there was no significant difference in bTMB among the three groups. In addition, utilizing the Catalog of Somatic Mutations in Cancer (COSMIC) mutation characteristic database (https://cancer.sanger.ac.uk/cosmic), it was discovered that HER2-0, HER2-low, and HER2-positive groups may exhibit distinct mutation signatures (Supplementary Fig. 2a–c).

### Mutation characteristics of HER2-low MBC in different HR status or different breast cancer molecular subtypes

**Fig. 2:**
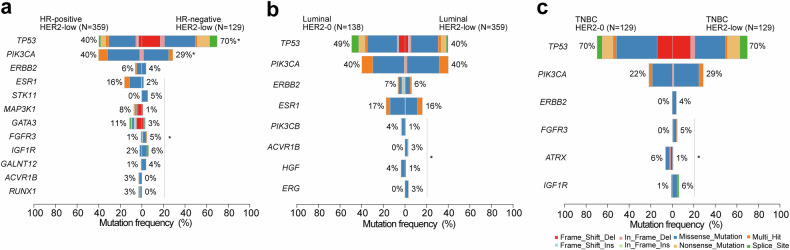
Comparison of mutation profiles in HER2-low MBC across different HR status or molecular subtypes. **a** Differential frequencies of gene mutations between HR-positive and HR-negative HER2-low MBC. **b**, **c** Differential gene mutation frequencies between luminal breast cancer and TNBC in the context of HER2-0 and HER2-low groups. *P < 0.05

The HR status plays a crucial role in the underlying biological characteristics of HER2-low breast cancer. In comparison to the HR-negative subgroup with HER2-low tumors, the mutation frequency of *ESR1*, *MAP3K1*, *GATA*, and *PIK3CA* was higher in the HR-positive subgroup. Conversely, *TP53*, *STK11*, *FGFR3*, and *IGF1R* exhibited lower mutation frequencies (Fig. 2a and Supplementary Fig. 3a, b). Mutant genes within the HR-positive subgroup were predominantly enriched in PI3K, ERBB2, and metabolic pathways, whereas mutations within the HR-negative subgroup were more concentrated in DNA-damage response (DDR) pathway (Supplementary Fig. 3c). The median bTMB for HR-positive and HR-negative HER2-low subgroups were 3.64 (range: 0.91–77.27) and 3.64 (range: 0.91–30.91), respectively (Supplementary Fig. 3d).

Furthermore, we investigated the specific characteristics of HER2-low breast cancer in different molecular subtypes of breast cancer. In luminal breast cancer, *PIK3CB*, *ACVR18*, *HGF*, and *ERG* were identified as the key genes with varying mutation frequencies between the luminal HER2-low and luminal HER2-0 subgroup (Fig. 2b). The major genes with distinct mutation frequencies in TNBC HER2-low and HER2-0 subtypes were *FGFR3*, *ATRX*, and *IGF1R* (Fig. 2c). Notably, all *ERBB2* mutations observed in TNBC (5/258) occurred exclusively within the TNBC HER2-low subgroup. Furthermore, no significant differences in bTMB were observed among the groups (Supplementary Fig. 3e, f).

### Evaluation of the efficacy of HER2-TKI in HER2-0 and HER2-low MBC patients with *ERBB2* mutation

**Fig. 3:**
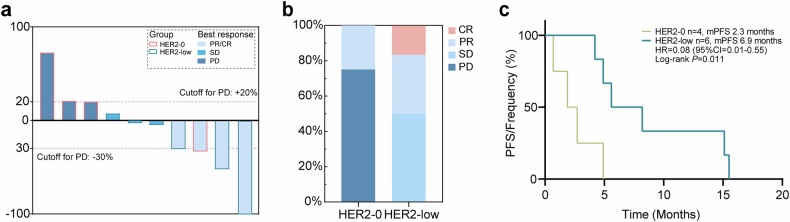
Clinical outcomes of pyrotinib therapy in HER2-0, HER2-low MBC patients with *ERBB2* mutations. **a** Optimal change in target lesions from baseline after treatment in HER2-0, HER2-low MBC patients with *ERBB2* mutations. **b** Distribution of the efficacy of pyrotinib therapy in HER2-0, HER2-low MBC patients with *ERBB2* mutations. **c** PFS analysis of HER2-0, HER2-low MBC patients with *ERBB2* mutations treated with pyrotinib

Previous studies have suggested the efficacy of HER2-TKI in HER2-negative patients with *ERBB2* mutation, including those with HER2-0 or HER2-low status. In this exploratory study, we conducted a small-scale analysis to the impact of HER2 status on the efficacy of pyrotinib treatment in patients with ctDNA-detected *ERBB2* mutations. This cohort of patients (cohort 2), all from a prospective phase 2 single-arm clinical trial (NCT03412383), included ten HER2-negative patients with *ERBB2* mutations, out of which six had HER2-low MBC, including one CR, two partial responses (PR), and three cases showed stable disease (SD). Additionally, there were four patients classified as HER2-0, among whom three experienced progressive disease (PD) and one achieved a PR (Fig. 3a). The disease control rate (DCR) and objective response rate (ORR) following treatment were higher in HER2-low MBC patients compared to those classified as HER2-0 MBC (Fig. 3b). Furthermore, an analysis was performed to evaluate PFS between both groups. It suggested that HER2-low group exhibited significantly prolonged PFS following pyrotinib treatment compared to the HER2-0 group (median PFS: 6.9 months vs 2.3 months, *P* = 0.011, Fig. 3c). The findings suggest that TKI treatment may be beneficial for HER2-low MBC patients among those with *ERBB2* mutations, while HER2-0 MBC patients are less likely to derive benefits from TKI treatment.

### Evaluation of the efficacy of chemotherapy, endocrine therapy, or CDK4/6 inhibitor therapy in HER2-0 and HER2-low MBC patients with metabolic pathway-related gene (MRG) mutations

**Fig. 4:**
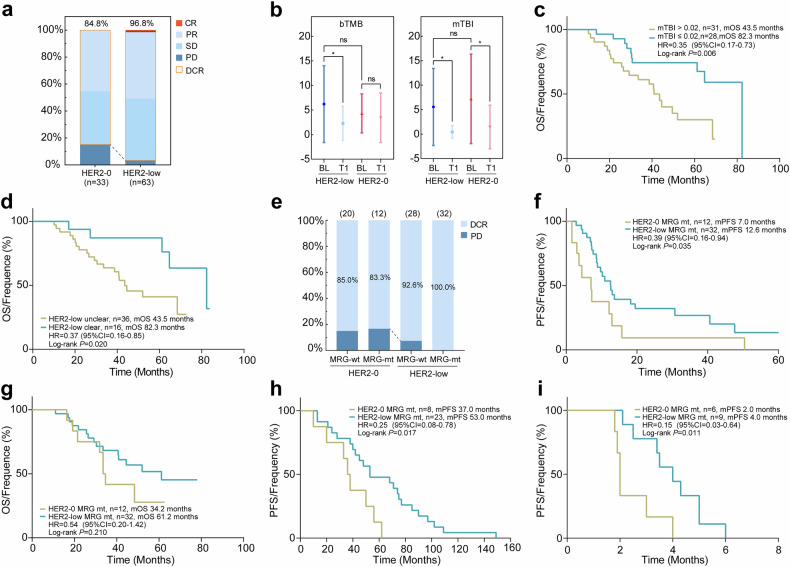
Survival analysis and clinical outcomes of HER2-0 and HER2-low MBC patients. **a** Assessment of the efficacy of TX regimen chemotherapy with HER2-0 and HER2-low MBC patients. **b** The bTMB and mTBI values of baseline (BL) and after the initial two treatment cycles (T1) of the groups. **c** OS analysis based on the mTBI after the initial two treatment cycles of HER2-low MBC. **d** OS analysis of HER2-low MBC patients with or without ctDNA cleared after the initial two treatment cycles. **e** The impact of HER2-0 and HER2-low MBC, with or without MRG mutations, on the alteration of DCR. **f**, **g** PFS/OS analysis of HER2-0 and HER2-low MBC with MRG mutations. **h** PFS analysis of HER2-0 and HER2-low MBC patients with MRG mutations who were treated with adjuvant endocrine therapy. **i** PFS analysis of HER2-0 and HER2-low MBC patients with MRG mutations who were treated with CDK4/6 inhibitor. *P < 0.05

The efficacy of chemotherapy for HER2-0 and HER2-low MBC in the TX regimen (docetaxel plus capecitabine) was initially assessed through a prospective phase 3 clinical trial (NCT01917279) utilizing ctDNA analysis. The cohort primarily comprised 63 HER2-low MBC patients and 33 HER2-0 MBC patients (cohort 3, Supplementary Table 1). Furthermore, we conducted an analysis on the clinicopathological characteristics of patients with HER2-0 or HER2-low MBC. We observed that the expression of Ki-67 was relatively lower in the HER2-low group compared to the HER2-0 group (*P* = 0.009, Supplementary Fig. 4a), while there were no significant differences in tumor load parameters such as tumor size and number of metastatic sites between the two groups. Additionally, there were no disparities in DFS results (Supplementary Fig. 4b–d). Subsequently, we analyzed the efficacy of HER2-0 and HER2-low MBC and found that the DCR of HER2-low MBC was higher than that of HER2-0 MBC (96.8% vs 84.9%, *P* = 0.045, Fig. 4a). However, both univariate and multivariate analyses showed no statistical difference in PFS and OS between the two groups (*P* > 0.05, Supplementary Fig. 4e, f and Supplementary Table 2). There was no significant difference in baseline bTMB between the HER2-0 and HER2-low groups, while the HER2-low group exhibited a more pronounced decrease at the first assessment (after the initial two treatment cycles). Previously, we identified that the molecular tumor burden index (mTBI) could serve as a mutational indicator of tumor burden,^24^ however, this association was not observed in the baseline values of the HER2-0 and HER2-low groups. Both groups showed a significant reduction in mTBI from baseline at the first assessment (Fig. 4b), and the mTBI value (cutoff = 0.02) at this assessment could potentially serve as a predictor for OS in HER2-low MBC patients (*P* = 0.006, Fig. 4c). Furthermore, analysis of ctDNA cleared during the first assessment revealed that patients who achieved ctDNA cleared had superior OS compared to those without ctDNA cleared (median OS: 82.3 months vs 43.5 months, *P* = 0.020, Supplementary Table 3 and Fig. 4d).

The previous analyses have found a higher prevalence of mutations in metabolic pathway in HER2-low MBC compared to HER2-0 MBC. We conducted further investigations on the survival outcomes between HER2-low MBC patients with MRG mutations and HER2-0 MBC patients with MRG mutations. Analysis of DCR suggested that a significantly higher DCR in the HER2-low group (Fig. 4e). Moreover, HER2-low group showed a longer PFS than HER2-0 group (median PFS: 12.6 months vs 7.0 months, *P* = 0.035, Fig. 4f). Our analysis of OS also found a more favorable trend in HER2-low MBC patients with MRG mutations compared to HER2-0 MBC, although this difference did not reach statistical significance (median OS: 61.2 months vs 34.2 months, *P* = 0.210, Supplementary Table 4 and Fig. 4g).

Furthermore, we investigated whether the two groups with MRG mutations exhibited similar patterns when subjected to other treatments. In the patients undergoing adjuvant endocrine therapy (AET), our analysis found no discernible disparity in the proportion of endocrine resistance between the HER2-0 and HER2-low groups (Supplementary Fig. 4g), as well as no significant distinction in PFS between these two groups (Supplementary Fig. 4h). Notably, HER2-low MBC group with MRG mutations exhibited superior PFS compared to HER2-0 group with MRG mutations (median PFS: 53.0 months vs 37.0 months, *P* = 0.017, Supplementary Table 5 and Fig. 4h).

In the cohort study investigating the efficacy of CDK4/6 inhibitor therapy (cohort 4), There was no discernible disparity in PFS between the cohorts of patients with HER2-0 MBC and HER2-low MBC who underwent palbociclib treatment (Supplementary Fig. 4i). However, patients with MRG mutations also exhibited a more favorable PFS trend within the HER2-low group as opposed to the HER2-0 group (median PFS: 4.0 months vs 2.0 months, *P* = 0.011, Fig. 4i).

### Identify molecular clusters of HER2-low MBC

**Fig. 5:**
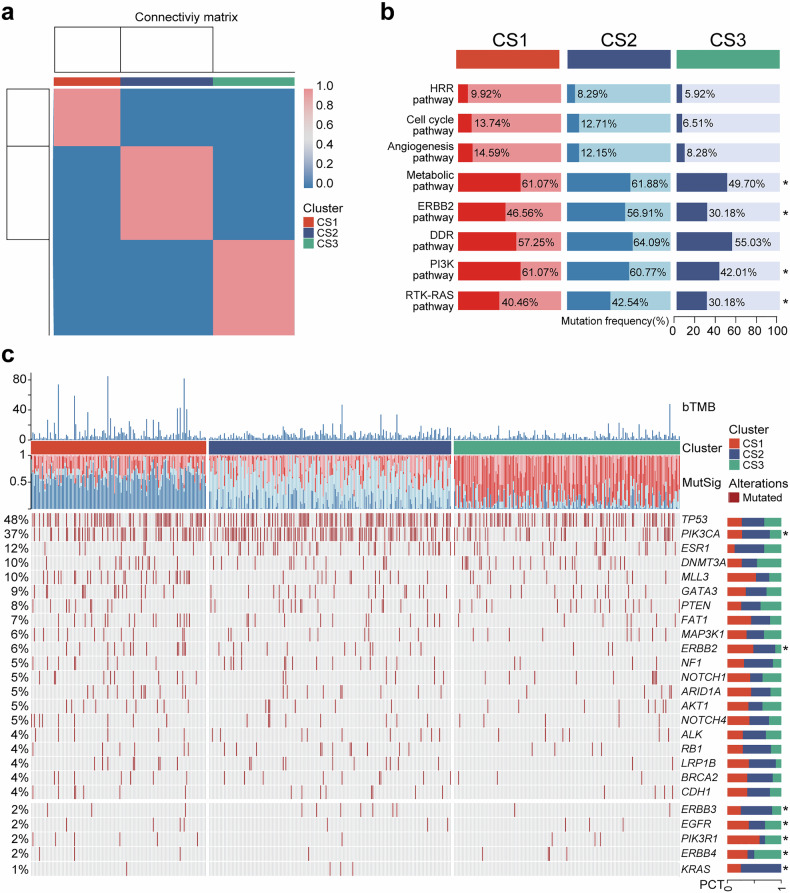
The clusters of HER2-low MBC based on plasma ctDNA. **a** Clustering of HER2-low breast cancer patients using NMF. **b** Characteristics of the three clusters in oncogenic signaling pathways. **c** Mutation landscape for three molecular clusters, including cluster 1 (CS1), cluster 2 (CS2), and cluster 3 (CS3), respectively. *P < 0.05

We conducted an analysis of the mutation signatures in cohort 1, comprising 488 HER2-low MBC patients. Utilizing a non-negative matrix factorization (NMF) algorithm, we determined the optimal rank value and independently identified three distinct clusters (Supplementary Fig. 5a and Fig. 5a). The mutation frequencies of RTK–RAS, PI3K, ERBB2, and metabolic pathways varied among the three clusters (Fig. 5b). Cluster 1 (CS1) exhibited a higher bTMB compared to cluster 2 (CS2) and cluster 3 (CS3) (Supplementary Fig. 5b). Further investigation revealed significant differences in the mutation signatures and molecular change spectra among these three molecular clusters. The mutation frequencies of *PIK3CA*, *ERBB2*, *ERBB3*, and *EGFR* genes varied across the three clusters. In addition, we observed the mutation frequency of some key resistance genes,^25,26^ for example, we found that the *ESR1* mutation frequency was low in CS1, and the *RB1* mutation frequency was low in CS3. Moreover, by aligning the mutation characteristics of drug-sensitive samples with the clustering patterns and mutational signatures observed in our study cohort, we found that the CS 1, 2, and 3 were more similar to those of treatment-sensitive samples receiving AET, TX regimens chemotherapy, and CDK4/6 inhibitor therapy, respectively (Supplementary Fig. 5c). The alterations in mutation spectrum observed within these clusters reflect heterogeneity within HER2-low MBC population, while also offering potential novel strategies for targeted therapies.

## Discussion

The outcomes of various clinical trials concerning ADCs, including DESTINY Breast04 and TROPICS02, provide solution to the treatment dilemma faced in HER2-negative breast cancer.^7,27^ Moreover, the potential of T-DXd in treating HER2-low MBC presents encouraging possibilities for tailored therapeutic strategies aimed at addressing HER2-low breast cancer. The main objective of this research was to examine the ctDNA genomic characteristics among individuals diagnosed with HER2-low MBC. By conducting a retrospective analysis on a cohort, we noted that the HER2-low group exhibited a higher prevalence of HR-positive patients compared to both the HER2-0 and HER2-positive groups. Consistent with previous studies, luminal breast cancers exhibit a greater frequency of HER2-low tumors in contrast to TNBC.^13,28–30^ The analysis of ctDNA from 1071 MBC patients revealed that among the 488 HER2-low MBC patients, *TP53*, *PIK3CA*, and *ESR1* exhibited the highest frequency of mutations. These findings align with previous, highlighting their consistent presence as the most common mutations.^9^ Compared to HER2-0 and HER2-positive patients, the incidence of *TP53* mutation is less frequent in HER2-low MBC patients, while the mutation frequency of *PIK3CA* and *ERBB2* falls between these two groups. Additionally, it appears that mutations in the *ERBB2* gene are more concentrated within the TKD site. Our previous study has confirmed that breast cancer patients with mutations at the *ERBB2* TKD site exhibit heightened sensitivity to anti-HER2 therapy, suggesting that such individuals may benefit more from this treatment approach.^31^ In terms of oncogenic pathways, the three groups of mutant genes primarily involved in the pathway were mainly related to RTK–RAS, ERBB2, and differences in metabolic or angiogenesis pathways. Furthermore, ~3% of TNBC patients exhibit *ERBB2* mutations. Interestingly, these mutations primarily occur within the HER2-low subgroup of TNBC.

Currently, HER2-targeted therapy has been expanded to HER2-low and even to HER2-ultra-low.^32^ Traditional anti-HER2 therapy may be less effective for HER2-low patients,^5,33^ but those with *ERBB2* mutations may still benefit from targeted therapy.^34^ The study investigated the influence of HER2 expression status on the effectiveness of pyrotinib. We observed that HER2-low MBC patients with *ERBB2* mutation may exhibit a more favorable response to pyrotinib, whereas HER2-0 patients with *ERBB2* mutations may display a less favorable response. The limited sample size of our study necessitates large-scale prospective studies to validate our findings. Therefore, we have scheduled additional investigations to assess the potential advantages of the HER2-ADC treatment for patients with *ERBB2* mutations. Most of the previous retrospective studies that received neoadjuvant chemotherapy or postoperative traditional therapy have shown different clinical characteristics and clinical outcomes of HER2-low breast cancer.^35–38^ We compared HER2-low and HER2-0 MBC patients receiving chemotherapy, and found no difference in PFS or OS between HER2-low and HER2-0 MBC patients. The dynamic analysis of ctDNA showed a more significant decrease in bTMB for HER2-low MBC after the second treatment cycle compared to HER2-0 MBC. Measurement of mTBI serves as a reliable predictor for treatment response and prognosis,^24,39^ and our findings indicate that mTBI can predict OS in HER2-low MBC patients. In addition, the results indicate that patients who achieve early clearance of ctDNA exhibit a more favorable prognosis. The identification of these prognostic markers associated with HER2-low MBC provides valuable insights for comprehensive patient evaluation.

Previous studies have suggested that HER2-low breast cancer may display distinct metabolic traits in contrast to HER2-0 tumors,^40,41^ suggesting a possible association between metabolism-related mutations and HER2-low breast cancer. Our study found, for the first time, that HER2-low patients with MRG mutations who received chemotherapy exhibited superior DCR and PFS compared to the HER2-0 group. In the studies pertaining to AET or CDK4/6 inhibitor therapy, there was no discernible difference in prognosis between HER2-0 and HER2-low group. Interestingly, HER2-low breast cancer patients with MRG mutations displayed a tendency towards improved PFS compared to HER2-0 breast cancer patients. Although these findings are exploratory due to limitations in sample size, they provide valuable insights for future treatment strategies targeting HER2-low breast cancer. The heterogeneity of HER2-low breast cancer is evident, and previous studies have attempted to characterize and classify it by molecular characteristics of primary tumor tissues.^21,41–43^ Due to the spatial and temporal heterogeneity of HER2, relying solely on primary tissue may not accurately reflect the molecular characteristics of MBC patients. Based on the aforementioned retrospective study, we investigated the classification of HER2-low MBC using ctDNA sequencing and initially identified three clusters that may offer guidance for treatment options. CS1 exhibits potential sensitivity to endocrine therapy, while CS2 may respond favorably to conventional chemotherapy. CS3 may exhibit a more favorable prognosis in terms of CDK4/6 inhibitor therapy.

Finally, it is worth noting that there exist certain additional constraints in relation to this study. Insufficient data regarding survival outcomes for cohort 1 and inadequate analysis of the influence on overall population’s survival among HER2-low patients were observed. The sample size of cohorts 2–4 is small and these results need to be further validated. Due to population characteristics, restricted data access, and other factors, we were unable to further validate these conclusions using external data. All of these aspects require further enhancement.

In conclusion, our study suggests that although HER2-low breast cancer should not be regarded as a separate molecular entity, the presence of ctDNA in MBC patients indicates that HER2-low MBC may possess certain unique characteristics. The efficacy of HER2-TKI in HER2-low patients with *ERBB2* mutations appeared to be potentially superior compared to HER2-0 MBC patients. Additionally, the incidence of MRG mutations was higher in HER2-low MBC compared to HER2-0 MBC. Among patients with MRG mutations, the prognosis of patients in the HER2-low group showed a tendency towards improvement upon receiving TX regimen chemotherapy, AET, or CDK inhibitor as opposed to HER2-0 group. Furthermore, early reduction in mTBI and early clearance of ctDNA may serve as prognostic indicators for HER2-low MBC. Considering the inherent heterogeneity of HER2-low MBC, a molecular classification system based on ctDNA was initially established in this study. The aforementioned findings serve as a reference for further comprehending HER2-low MBC and exploring personalized treatment strategies for these patients.

## Materials and methods

### Patients and sample collection

Cohort 1 was derived from 1184 patients with MBC. Specific inclusion and exclusion criteria were described previously.^31^ The status of HER2 was determined based on IHC and/or ISH. A total of 1071 patients with definite HR and HER2 status were finally included in this cohort. Cohort 2 comprises patients enrolled in our single-arm prospective phase 2 clinical trial (NCT03412383) investigating the efficacy of pyrotinib in individuals with *ERBB2*-mutated MBC. The specific inclusion and exclusion criteria remain unchanged.^31^ Cohort 3 was based on the prospective, randomized, phase 3 clinical trial (CAMELLIA, NCT01917279) involving HER2-negative MBC patients who received a combination of docetaxel and capecitabine at 32 clinical centers in China.^44^ After excluding 29 patients with uncertain HER2 IHC or ISH status, a cohort consisting of 96 patients was enrolled in the study. ctDNA sequencing data from pretreatment samples were used to explore molecular characteristics. DFS was defined as the time from diagnosis to disease recurrence/progression or last follow-up. PFS was defined as the duration from enrollment or treatment initiation to the occurrence of disease progression or death. OS was defined as the duration from the date of enrollment to the occurrence of death. Tumor response was assessed on imaging every two treatment cycles according to Response Evaluation Criteria in Solid Tumors (RECIST), version 1.1. The definition of DCR included the best overall response as CR, PR, or SD and the definition of ORR includes CR, PR. A total of 64 patients in cohort 3 who underwent standard AET (selective ER modulators or aromatase inhibitors) were included in the subsequent study. The duration of AET until disease progression was assessed with emphasis. Endocrine resistance was defined as relapse within 2 years of initiating AET. Cohort 4 consisted of patients who received CDK4/6 inhibitor (palbociclib) therapy at the Cancer Hospital of the Chinese Academy of Medical Sciences from June 2017 to June 2022. Excluding patients with a history of receiving more than three lines of advanced therapy or uncertain HER2 status, a total of 23 patients who had previously undergone two to three lines of therapy were included in this study.

The patients in this study all provided written consent and were approved by the Ethics Committee of the Cancer Hospital, Chinese Academy of Medical Sciences.

### Identification of somatic mutations in ctDNA samples

The somatic SNVs and small InDels of ctDNA sequencing data were analyzed using realDcaller (Geneplus-Beijing) and GATK Mutect2,^45^ an internally developed software for reviewing hotspot variants. To be considered a candidate somatic mutation in ctDNA samples, the following criteria were applied: (i) absence in matched peripheral blood monocyte cell DNA or a frequency more than double that of normal control WBCs; (ii) absence in >1% of the population according to the 1000 Genomes Project or dbSNP databases; (iii) no evidence of sequencing strand bias or template strand bias; (iv) no concentration on one side of the reads; (v) positional mutation depth exceeding 300×; (vi) further filtering based on recurrent artifacts derived from plasma samples of healthy individuals, removing mutations with frequencies not significantly higher than those in the baseline background database; and finally, (vii) presence of ≥4 high-quality support reads for hotspot mutations or ≥8 support reads for non-hotspots.

### Clonal hematopoiesis mutation filtering process

Clonal hematopoiesis (CH) mutations are meticulously filtered through comprehensive deep sequencing of white blood cells derived from matched blood samples. The variant allele frequencies of CH mutations identified in cell-free DNA are systematically correlated with their corresponding VAFs in the paired blood samples. Specifically, any mutation frequency observed in ctDNA that does not exceed twice the frequency detected in the paired blood sample is designated and filtered as a CH mutation. Furthermore, a CH mutation blacklist has been meticulously compiled based on extensive published data. This blacklist serves as an additional criterion for filtering CH mutations, ensuring that these are also screened against the blacklist in conjunction with the analysis of the paired blood samples. This dual-filtering approach enhances the precision and reliability of the CH mutation identification process.

### Genomic analysis of ctDNA

The patient blood samples were collected, extracted, and processed for sequencing following the manufacturer’s instructions and previous protocols.^24,31^ Briefly, we collected peripheral blood samples from each patient and isolated ctDNA from plasma using the QIAamp Circulating Nucleic Acid Kit (Qiagen, CA, USA). Following the specified criteria, we performed polymerase chain reaction amplification on eligible samples. To detect 1021 common mutant genes, we hybridized the library with biotinylated oligonucleotide probes (Integrated DNA Technologies, MI, USA). For DNA sequencing analysis, we utilized the Gene+seq2000 sequencing system (Geneplus, Suzhou, China). The gene set of oncogenic pathways (RTK–RAS, PI3K and Cell cycle,^46^ ERBB2,^47^ Metabolic,^48^ Angiogenesis, DDR and HRR pathways^49^) were obtained from previous publications. The bTMB was calculated by dividing the cumulative number of mutations identified in plasma ctDNA samples from each patient by the overall length of the panel’s coding region (1.1 MB). The definition of mTBI involves calculating the cumulative mutational allele frequencies derived from unique variations identified in a series of plasma ctDNA samples, while considering heterogeneity and dynamic evolution.

### Identification of clusters and analysis of drug reactivity associations

The molecular clustering and its relationship with drugs were explored by employing the principle of mutation signature similarity.^50^ Specifically, we utilized the “maftools” R package and “trinucleotideMatrix”, “estimateSignature”, and “extractSignature” functions to accurately extract de-novo signature. The determination of the optimal number of signatures (2~6) was based on cophenetic correlation metric, enabling us to establish precise mutation-related signatures. Subsequently, we employed NMF to efficiently aggregate tumor samples by combining common clusters and consensus values across different levels (2~10). Our analysis successfully identified three distinct clusters in HER2-low MBC samples. Furthermore, we extracted mutation signatures from gene sets of treatment-sensitive cohorts of chemotherapy, endocrinology and CDK4/6 inhibitor therapy and subsequently developed customized signatures accordingly. Finally, the cosine similarity between the mutated signatures and the selected signatures in the target queue is calculated utilizing a custom script based on the “cosine” function.

### Statistical analysis

The survival outcomes of MBC patients in different groups were assessed using Kaplan–Meier curves and two-sided log-rank tests. Potential confounders were adjusted for through univariate and multifactor COX regression analyses. Fisher’s exact tests were employed to compare the proportional composition of two or more variables. The *t*-test or one-way ANOVA analysis of variance was used to compare sets of continuous variables, while Mann–Whitney test or Kruskal–Wallis test was utilized for ordered categorical variables comparison. All statistical analyses were conducted using SPSS 22.0, GraphPad Prism 8.0, and R 4.3.3 software packages. The criterion for statistical significance was set at a *P* value < 0.05.

## Supplementary information

Study Protocol

Supplementary Materials

## Data Availability

All data supporting the findings of this research can be obtained upon a reasonable inquiry.
