# Supplementary material for: Genomic landscape of circulating tumor DNA in HER2-low metastatic breast cancer
Source: Signal Transduct Target Ther. 2024 Dec 9;9:345. doi: 10.1038/s41392-024-02047-0 (PMC11625825; doi:10.1038/s41392-024-02047-0)
Supplement: Supplementary file 1 — Study Protocol [file 41392_2024_2047_MOESM1_ESM.doc]

# A Phase II Study of Pyrotinib in Metastatic HER2 Non-amplified But HER2 Mutant Breast Cancer

(Investigator initiated trial)

**PROTOCOL**

**Version:** 2.0

**Dated:** 13 December 2017

**Chief Investigator:** Prof Fei Ma

**ClinicalTrials.gov Identifier:** NCT03412383

**Sponsor:** National Cancer Center/National Clinical Research Center for Cancer/Cancer Hospital, Chinese Academy of Medical Sciences and Peking Union Medical College

**CONTENTS**

[**1. Study Official Title 3**](#__RefHeading___Toc40184363)

[**2. Background 3**](#__RefHeading___Toc40184364)

[**3. Purpose 3**](#__RefHeading___Toc40184365)

[Primary Outcome Measures: 4](#__RefHeading___Toc40184366)

[Secondary Outcome Measures: 4](#__RefHeading___Toc40184367)

[**4. Study Design 4**](#__RefHeading___Toc40184368)

[**5. Inclusion Criteria: 4**](#__RefHeading___Toc40184369)

[**6. Exclusion Criteria: 5**](#__RefHeading___Toc40184370)

[**7. Sample collection and DNA extraction 5**](#__RefHeading___Toc40184371)

[**8. Target capture and next-generation sequencing 6**](#__RefHeading___Toc40184372)

[**9. Sequencing data analysis 6**](#__RefHeading___Toc40184373)

[**10. Treatment 7**](#__RefHeading___Toc40184374)

[**References: 7**](#__RefHeading___Toc40184375)

1. **Study Official Title**

A Phase II Study of Pyrotinib in Metastatic HER2 Non-amplified But HER2 Mutant Breast Cancer

1. **Background**

Breast cancer, one of the most common cancers worldwide in women, is a heterogeneous disease with a variety of outcomes and drug responses. The St. Gallen subtype classification, introduced in 2011, categorizes breast cancer into five basic therapeutic groups based on immunohistochemical staining. These subtypes include luminal A, luminal B HER2-neu negative, luminal B HER2-neu positive, HER2-neu non-luminal and basal-like. Human epidermal growth factor receptor 2 (HER2, also known as ERBB2) amplification or overexpression is found in 20-30% of breast cancer patients and is associated with poor prognosis ^1,2^.

HER2 protein overexpression or gene amplification is an important predictive biomarker for identifying breast cancer patients who may benefit from anti-HER2-targeted therapy. However, little is known about the effects of HER2 mutations on anti-HER2 therapy in advanced breast cancer patients. Several studies have indicated that the mechanisms of HER2 activation include not only HER2 protein overexpression and gene amplification but also somatic mutations in HER2, leading to activation of the HER2 gene ^3,4^. Next-generation sequencing has indicated that somatic mutations in HER2 are found in approximately 2-5% of primary breast cancers ^5-7^. Most HER2 somatic mutations have been reported in HER2-negative breast cancers ^8^.

We conducted a phase-II clinical trial to explore the efficacy of pyrotinib in HER2 amplification-negative mutation-positive metastatic breast cancer patients.

1. **Purpose**

To explore the efficacy and safety of pyrotinib in HER2 amplification-negative mutation-positive metastatic breast cancer patients.

## Primary Outcome Measures:

Progression-free survival (PFS)

From date of first use Pyrotinib until the date of first documented progression or date of death from any cause, whichever came first.

## Secondary Outcome Measures:

- **Adverse events (AEs):** Adverse events (AEs) and laboratory tests graded according to the NCI CTCAE (version 4.0)
- **Overall Response rates (ORR):** Defined as complete response (CR) + partial response (PR), assessed based on Response Evaluation Criteria in Solid Tumors (RECIST 1.1) criteria.
- **Clinical Benefit rate (CBR):** Defined as CR+PR+stable disease (SD) over 24 weeks, assessed based on on Response Evaluation Criteria in Solid Tumors (RECIST 1.1) criteria.
- **Time to Progression (TTP):** Time from first use Pyrotinib to disease progression
- **Quality of Life (QoL):** Using the EORTC quality of life questionnaire QLQ-C30
- **Overall survival (OS):** Time from first use Pyrotinib to death

1. **Study Design**

Study Type: Interventional (Clinical Trial)

Estimated Enrollment: 14 participants

Intervention Model: Single Group Assignment

Masking: None (Open Label)

1. **Inclusion Criteria:**

- Able to understand and willing to sign an IRB approved written informed consent document.
- At least 18 years of age.
- Histologically or cytologically confirmed HER2-negative (0 or 1+ by IHC or non-amplified by FISH) breast cancer that is stage IV.
- There is no standard therapy.
- At least one measurable disease by RECIST 1.1 is required.
- KPS>70, life expectancy > 12 weeks

1. **Exclusion Criteria:**

- Lack of adequate organ function as defined below within 2 weeks of registration:

ANC<1.5×10^9/L，PLT<75×10^9/L or Hb<100g/L

TBiL>2×ULN; AST or ALT>2.5×ULN（or>5 x ULN for patients with liver metastases）; ALP>2.5×ULN; Scr>140umol/L

- Pregnant and/or breastfeeding.
- History of significant cardiac disease, cardiac risk factors, or uncontrolled arrhythmias.
- Having a history of uncontrolled paroxysmal diseases, including central nervous system diseases or mental disorders which may have an impact on the understanding and signature of informed consent
- Uncontrolled acute infection
- Currently receiving any other investigational agents or systemic cancer therapy.
- Allergy to any investigational drug;
- Any other condition that investigator considers inappropriate to participate in this trail

1. **Sample collection and DNA extraction**

Ten ml peripheral blood was collected from each patient. Peripheral blood samples were collected in Streck tubes (Streck, Omaha, NE, USA) and centrifuged within 72 hours to separate the plasma from the peripheral blood cells. All methods were performed in accordance with the relevant guidelines and regulations, and informed consent was obtained from all patients.

Circulating DNA was isolated by using QIAamp Circulating Nucleic Acid Kits (Qiagen, Hilden, Germany). Genomic DNA from tissues or peripheral lymphocytes was isolated using the DNeasy Tissue Kit, QIAamp DNA Kit and QIAamp DNA Blood Mini Kit, respectively. All DNA extractions were performed according to the manufacturer’s protocols, and genomic DNA from lymphocytes was sequenced as the normal control sample.

# 8. Target capture and next-generation sequencing

Sequencing libraries were prepared for cfDNA using the KAPA DNA Library Preparation Kit (Kapa Biosystems, Wilmington, MA, USA), and gDNA sequencing libraries were prepared using the protocols recommended in the Illumina TruSeq DNA Library Preparation Kit (Illumina, San Diego, CA). For samples at or near the minimum input requirement, additional pre-capture PCR cycles were performed to generate sufficient PCR product for hybridization. Libraries were hybridized to custom-designed biotinylated oligonucleotide probes (Roche NimbleGen, Madison, WI, USA) covering ~1.1 Mbp of sequence. DNA sequencing was carried out with the HiSeq 3000 Sequencing System (Illumina, San Diego, CA) with 2×101-bp paired-end reads.

1. **Sequencing data analysis**

After removing the terminal adaptor sequences and low-quality data, the reads were mapped to the reference human genome. GATK (https://www.broadinstitute.org/gatk/, The Genome Analysis Toolkit) and MuTect were used to call small insertions and deletions (indels) and single nucleotide variants (SNVs) in the somatic DNA by filtering peripheral blood (PBL) sequencing data. In addition, we used the NoahCare Tool Kit using NCfilter (software developed by self, version 1.5.0) for fastq data QC, NCbamInfo (version 0.2.0) for alignment QC; NCanno (version 0.1.1) for annotation with multiple databases; and NChot (version 0.1.0) for hotspot region variant review and recall. Contra was used to detect copy number variants, and BreakDancer was used to detect cancer-associated structural variants. The final candidate variants were all manually verified using the Integrative Genomics Viewer (IGV) Browser.

1. **Treatment**

The patients detected HER2 somatic mutations enrolled and received 400 mg of pyrotinib per day. The efficacy assessment was based on the Response Evaluation Criteria in Solid Tumors (RECIST) v1.1. Adverse events (AEs) were assessed according to the National Cancer Institute Common Terminology Criteria for Adverse Events version 4.0.

# References:

1. Owens MA, Horten BC, Da Silva MM: HER2 amplification ratios by fluorescence in situ hybridization and correlation with immunohistochemistry in a cohort of 6556 breast cancer tissues. Clin Breast Cancer 5:63-9, 2004

2. Yaziji H, Goldstein LC, Barry TS, et al: HER-2 testing in breast cancer using parallel tissue-based methods. JAMA 291:1972-7, 2004

3. Khoury T, Mojica W, Hicks D, et al: ERBB2 juxtamembrane domain (trastuzumab binding site) gene mutation is a rare event in invasive breast cancers overexpressing the ERBB2 gene. Mod Pathol 24:1055-9, 2011

4. Bose R, Kavuri SM, Searleman AC, et al: Activating HER2 mutations in HER2 gene amplification negative breast cancer. Cancer Discov 3:224-37, 2013

5. Endo Y, Dong Y, Yoshimoto N, et al: HER2 mutation status in Japanese HER2-negative breast cancer patients. Jpn J Clin Oncol 44:619-23, 2014

6. Boulbes DR, Arold ST, Chauhan GB, et al: HER family kinase domain mutations promote tumor progression and can predict response to treatment in human breast cancer. Mol Oncol 9:586-600, 2015

7. Zuo WJ, Jiang YZ, Wang YJ, et al: Dual Characteristics of Novel HER2 Kinase Domain Mutations in Response to HER2-Targeted Therapies in Human Breast Cancer. Clin Cancer Res 22:4859-4869, 2016

8. Zehir A, Benayed R, Shah RH, et al: Mutational landscape of metastatic cancer revealed from prospective clinical sequencing of 10,000 patients. Nat Med 23:703-713, 2017

# A Randomized Phase III Study of Metronomic vs. Intermittent Capecitabine Maintenance Therapy Following First-line Capecitabine and Docetaxel Therapy in HER2-negative Metastatic Breast Cancer

**Chief Investigator:** Prof Binghe Xu

**ClinicalTrials.gov Identifier:** NCT01917279

**Sponsor:** Binghe Xu

**Information provided by** Binghe Xu, Chinese Academy of Medical Sciences (Responsible Party)

The detailed PROTOCOL can be found on the following link: https://clinicaltrials.gov/study/NCT01917279.
