# Supplementary material for: Genomic landscape of circulating tumor DNA in HER2-low metastatic breast cancer
Source: Signal Transduct Target Ther. 2024 Dec 9;9:345. doi: 10.1038/s41392-024-02047-0 (PMC11625825; doi:10.1038/s41392-024-02047-0)
Supplement: Supplementary file 2 — Supplementary Materials [file 41392_2024_2047_MOESM2_ESM.docx]

Supplementary Materials for

Genomic landscape of circulating tumor DNA in HER2-low metastatic breast cancer

Zongbi Yi, Kaixiang Feng, Dan Lv, Yanfang Guan, Youcheng Shao, Fei Ma, Binghe Xu

Correspondence to: [xubh@cicams.ac.cn or](mailto:xubh@cicams.ac.cn%20or) [drmafei@126.com](mailto:drmafei@126.com)

**This PDF file includes:**

Figures. S1 to S5

Tables S1 to S5

Figure. S1.
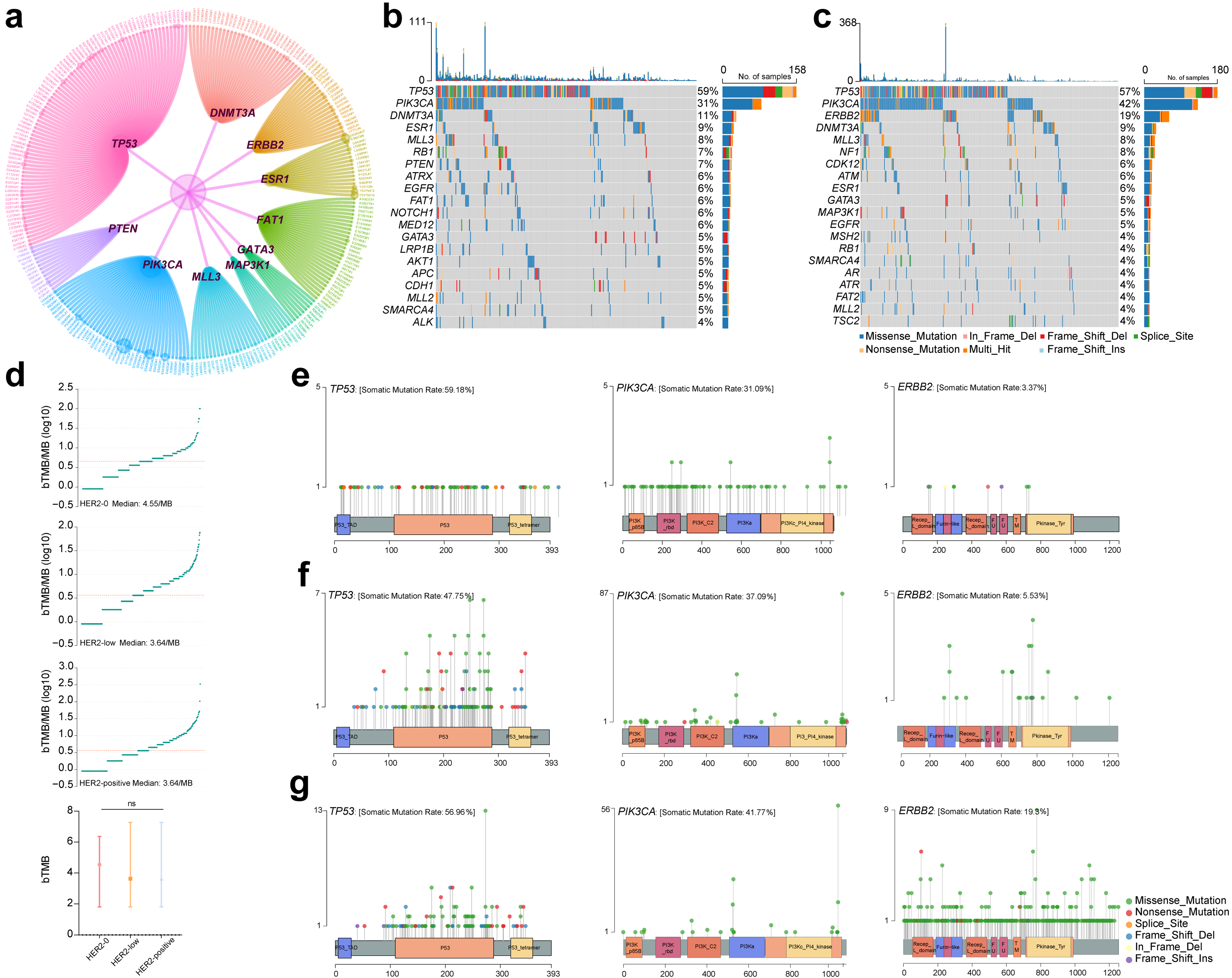


**Figure. S1. Mutations in different HER2 groups of MBC.**

(a). Top 10 genes with hot mutations in HER2-low MBC and their corresponding mutation sites. (b, c). Mutation profiles of HER2-0 and HER2-positive MBC. (d). Comparison of blood tumor mutation burden (bTMB) among three groups. (e-g). Mutation sites distribution of *TP53*, *PIK3CA*, and *ERBB2* genes in HER2-0, HER2-low, and HER2-positive MBC.

Figure. S2.
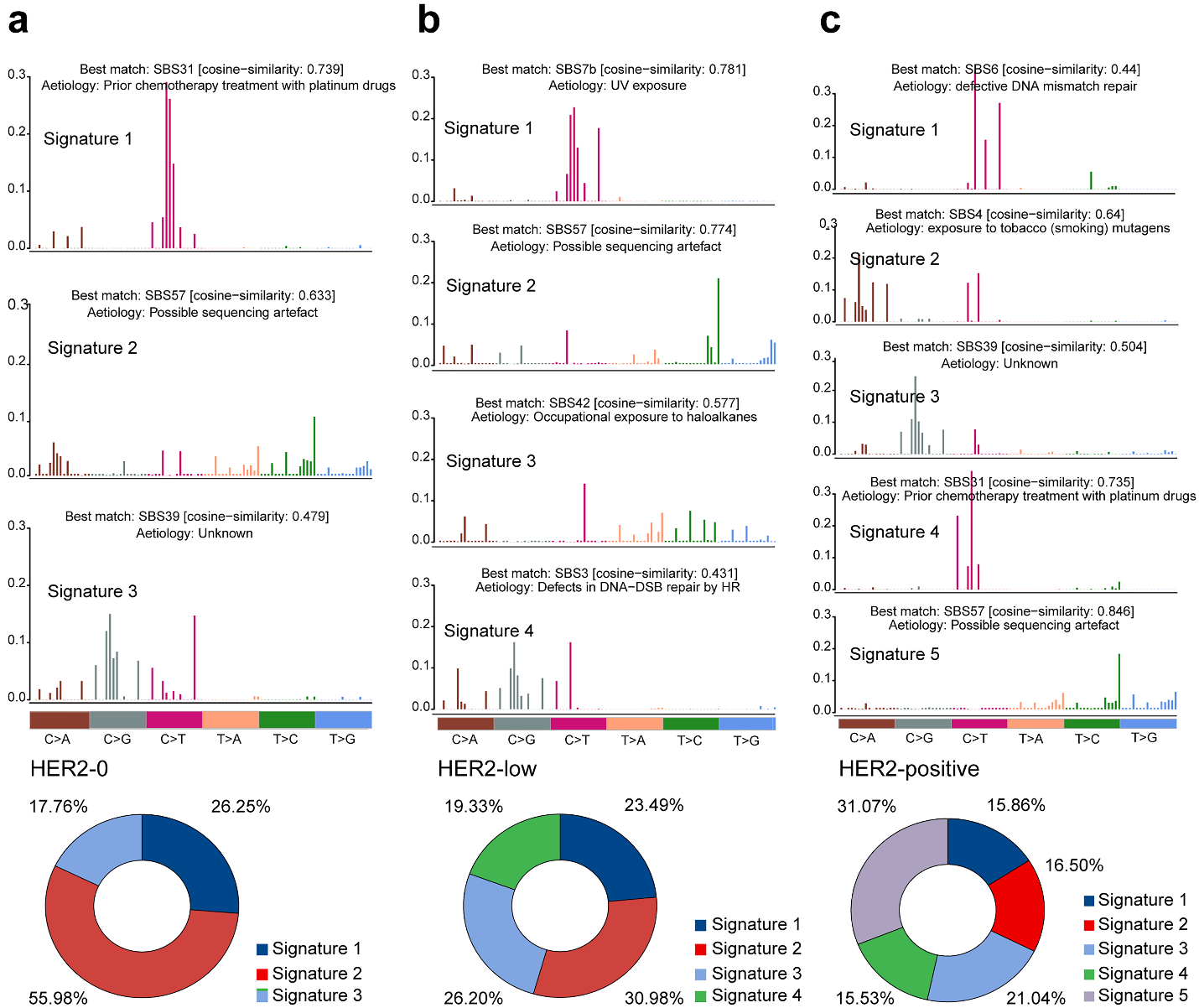


**Figure. S2. Mutation signatures of different HER2 groups in MBC.**

Mutation signatures in the (a) HER2-0, (b) HER2-low, and (c) HER2-positive groups were identified by leveraging signatures available in the COSMIC database.

Figure. S3.
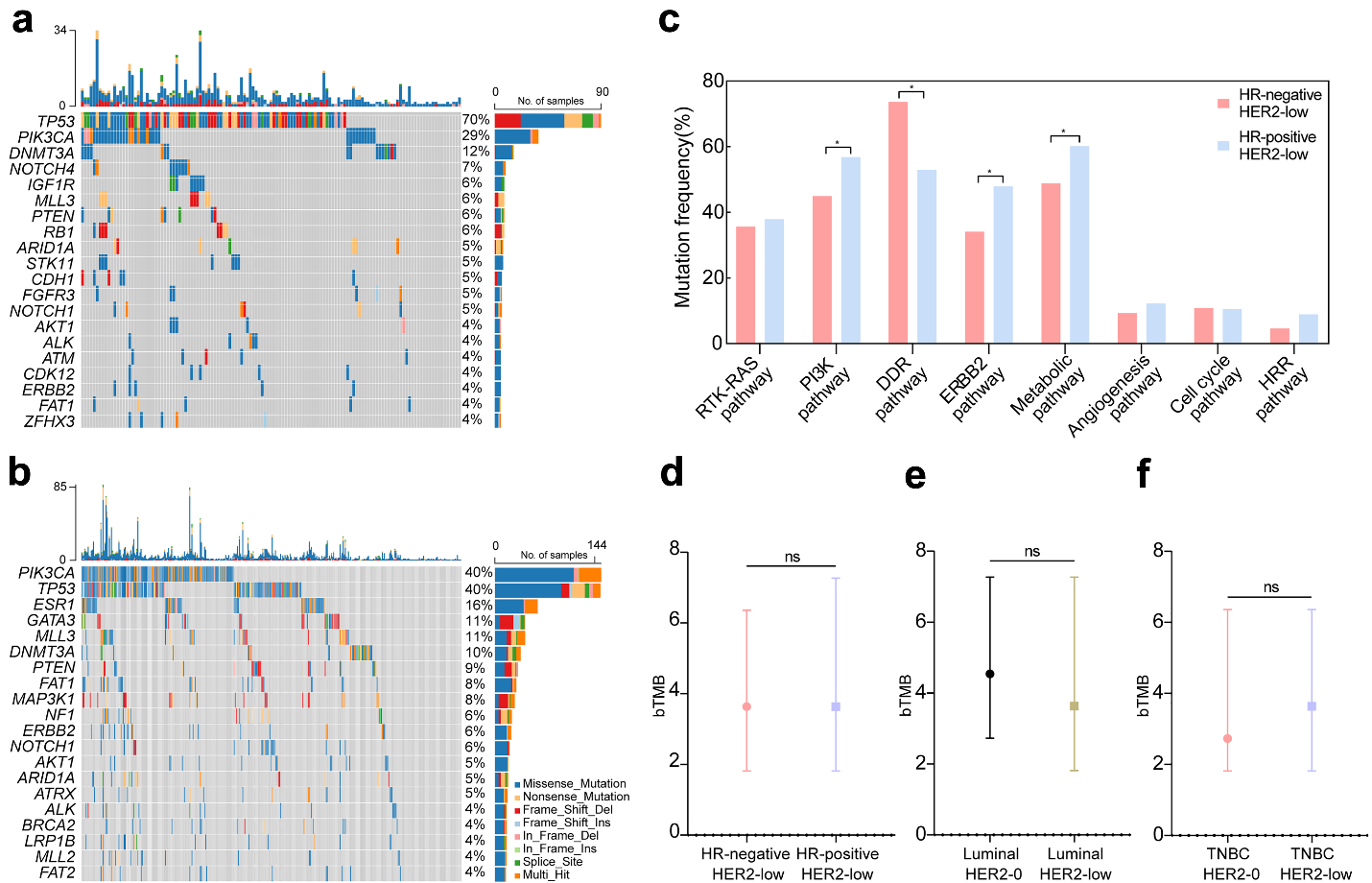


**Figure. S3. Mutation characters of** **HER2-low MBC across distinct subgroups of breast cancer.**

(a, b). Mutation profiles of HR-negative and HR-positive HER2-low MBC. (c). Characteristics analysis of common oncogenic signaling pathways in HR-positive and HR-negative HER2-low MBC. (d). Comparison of bTMB between HR-positive and HR-negative HER2-low MBC. (e). Comparison of bTMB between HER2-0 and HER2-low groups in luminal breast cancer. (f). Comparison of bTMB between HER2-0 and HER2-low groups in TNBC.

Figure. S4.
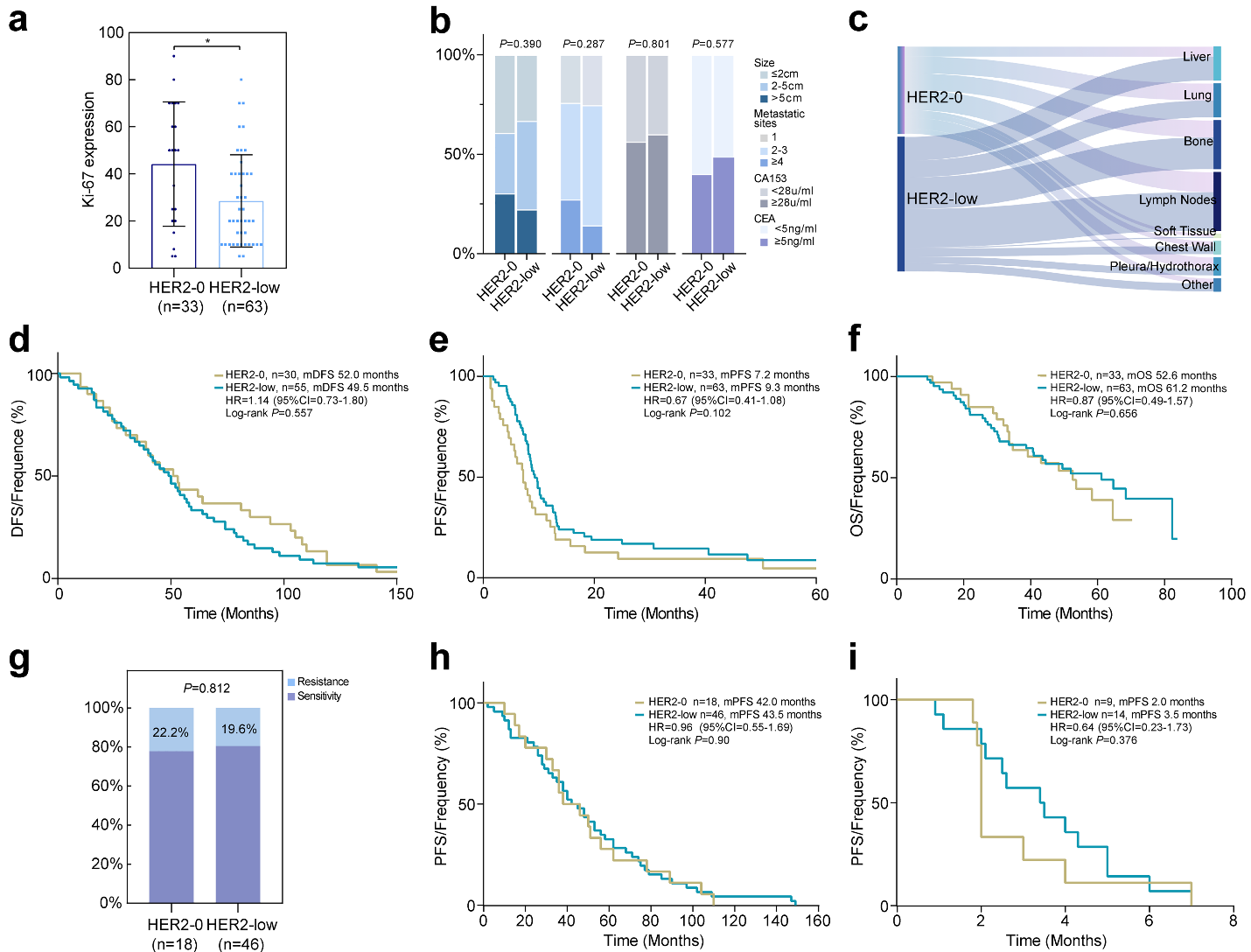


**Figure. S4. Clinical features and prognosis of HER2-0 and HER2-low MBC treated with drugs.**

(a). The Ki-67 levels in patients of HER2-0 and HER2-low group. (b). The characteristics of tumor size, number of metastatic sites, as well as the distribution patterns of CA153 and CEA, compared between patients with HER2-0 and HER2-low MBC. (c). Tumor metastasis in patients with HER2-0 and HER2-low MBC. (d). DFS analysis of HER2-0 and HER2-low MBC. (e-f). PFS/OS analysis of HER2-0 and HER2-low MBC. (g). Proportion of resistant patients with HER2-0 and HER2-low breast cancer who received adjuvant endocrine therapy. (h). PFS analysis of HER2-0 and HER2-low breast cancer patients who received adjuvant endocrine therapy. (i). PFS analysis of HER2-0 and HER2-low breast cancer patients who received CDK4/6 inhibitor therapy. * *P* < 0.05.

Figure. S5.
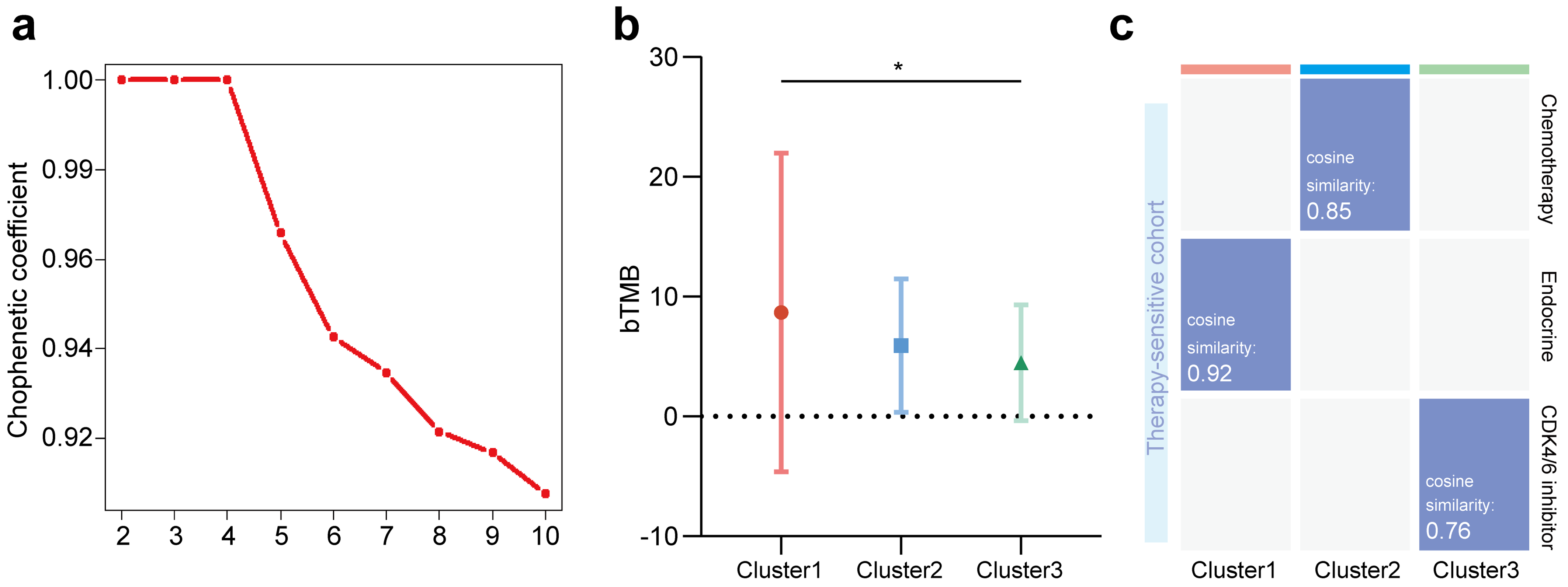


**Figure. S5. Three clusters of HER2-low MBC.**

(a). The cluster prediction index for clusters. (b). Comparison of bTMB between the three clusters. (c). Similarity of mutation signatures in different clusters to those in the TX regimen chemotherapy, adjuvant endocrine therapy, and CDK4/6 inhibitor therapy treatment-sensitive cohorts.

Table S1. Patient characteristics of cohort 3.

| Characteristics | NO. of patients (%) | | |
| --- | --- | --- | --- |
|  | Total  (n=96) | HER2-zero  (n=33) | HER2-low  (n=63) |
| Age |  |  |  |
| ≤35 | 17 | 3 (6.1) | 14 (22.2) |
| 35-60 | 76 | 29 (87.9) | 47 (74.6) |
| ＞60 | 3 | 1 (3.0) | 2 (3.2) |
| Histopathological |  |  |  |
| Ductal | 81 | 28 (84.9) | 53 (84.1) |
| Lobular | 7 | 4 (12.1) | 3 (4.8) |
| Mixed | 2 | 0 | 2 (3.2) |
| Other | 6 | 1 (3.0) | 5 (7.9) |
| Size-base line |  |  |  |
| ≤2cm | 34 | 13 (39.4) | 21 (33.3) |
| 2-5cm | 38 | 10 (30.3) | 28 (44.5) |
| ＞5cm | 24 | 10 (30.3) | 14 (22.2) |
| HER2 status |  |  |  |
| IHC 0 | 33 | 33 (100.0) | 0 |
| IHC 1+ | 37 | 0 | 37 (58.7) |
| IHC 2+/ISH - | 26 | 0 | 26 (41.3) |
| HR status |  |  |  |
| Positive | 85 | 27 (81.8) | 58 (92.1) |
| Negative | 11 | 6 (18.2) | 5 (7.9) |
| Grade |  |  |  |
| 1 | 2 | 1 (3.0) | 1 (1.6) |
| 2 | 48 | 18 (54.6) | 30 (47.6) |
| 3 | 15 | 6 (18.2) | 9 (14.3) |
| Unknown | 31 | 8 (24.2) | 23 (36.5) |
| Tumor stage |  |  |  |
| 1-2 | 50 | 19 (57.6) | 31 (49.2) |
| 3-4 | 34 | 11 (33.3) | 23 (36.5) |
| Unknown | 12 | 3 (9.1) | 9 (14.3) |
| Metastatic sites |  |  |  |
| 1 | 24 | 8 (24.2) | 16 (25.4) |
| 2-3 | 54 | 16 (48.5) | 38 (60.3) |
| ≥4 | 18 | 9 (27.3) | 9 (14.3) |

*HER2 human epidermal growth factor receptor 2, IHC immunohistochemical staining, ISH in situ hybridization, HR hormone receptor.*

Table S2. Cox regression analyses between PFS/OS and clinical characteristics of cohort 3.

Table S2.1. Cox regression analyses of PFS in cohort 3.

| **Variables** | **Univariate COX** | | **Multivariable COX** | |
| --- | --- | --- | --- | --- |
|  | HR (95% CI) | *P* | HR (95% CI) | *P* |
| HER2 status |  |  |  |  |
| HER2-low vs. HER2-0 | 0.69 (0.44-1.08) | 0.106 | 0.79 (0.49-1.27) | 0.333 |
| Hormone receptor |  |  |  |  |
| Positive vs. Negative | 0.69 (0.34-1.37) | 0.286 | 0.44 (0.20-0.94) | 0.034 |
| Bone metastasis |  |  |  |  |
| Yes vs. No | 0.60 (0.39-0.93) | 0.021 | 0.47 (0.30-0.74) | 0.001 |
| First-line endocrine therapy |  |  |  |  |
| Yes vs. No | 1.82 (1.12-2.93) | 0.016 | 2.43 (1.43-4.20) | 0.001 |
| Radiation therapy |  |  |  |  |
| Yes vs. No | 1.48 (0.95-2.29) | 0.081 | 1.53 (0.95-2.44) | 0.078 |

Table S2.2. Cox regression analyses of OS in cohort 3.

| **Variables** | **Univariate COX** | | **Multivariable COX** | |
| --- | --- | --- | --- | --- |
|  | HR (95% CI) | *P* | HR (95% CI) | *P* |
| HER2 status |  |  |  |  |
| HER2-low vs. HER2-0 | 0.88 (0.50-1.56) | 0.657 | 0.97 (0.53-1.77) | 0.914 |
| Hormone receptor |  |  |  |  |
| Positive vs. Negative | 0.84 (0.36-1.97) | 0.686 | 0.72 (0.28-1.83) | 0.488 |
| Liver metastasis |  |  |  |  |
| Yes vs. No | 1.54 (0.88-2.69) | 0.127 | 1.53 (0.83-2.84) | 0.173 |
| Number of metastatic organs |  |  |  |  |
| 2-3 vs. 1 | 1.37 (0.66-2.81) | 0.399 | 1.37 (0.66-2.82) | 0.396 |
| ≥4 vs. 1 | 1.96 (0.85-4.56) | 0.117 | 1.66 (0.68-4.05) | 0.261 |

Table S3. Cox regression analyses between OS and clinical characteristics of HER2-low MBC patients after the initial two treatment cycles.

Table S3.1. Cox regression analyses of OS in patients with different mTBI.

| **Variables** | **Univariate COX** | | | **Multivariable COX** | |
| --- | --- | --- | --- | --- | --- |
|  | HR (95% CI) | | *P* | HR (95% CI) | *P* |
| mTBI |  | |  |  |  |
| high vs. low | 2.96 (1.33-6.59) | | 0.008 | 3.03 (1.32-6.94) | 0.009 |
| Hormone receptor |  | |  |  |  |
| Positive vs. Negative | 0.62 (0.19-2.07) | | 0.435 | 0.61 (0.17-2.24) | 0.459 |
| Liver metastasis |  |  | |  |  |
| Yes vs. No | 2.06 (0.99-4.28) | | 0.053 | 2.40 (1.10-5.26) | 0.029 |

Table S3.2. Cox regression analyses of OS in patients with ctDNA cleared or uncleared.

| **Variables** | **Univariate COX** | | | **Multivariable COX** | |
| --- | --- | --- | --- | --- | --- |
|  | HR (95% CI) | | *P* | HR (95% CI) | *P* |
| ctDNA |  | |  |  |  |
| cleared vs. uncleared | 0.29 (0.10-0.87) | | 0.027 | 0.32 (0.10-0.96) | 0.041 |
| Hormone receptor |  | |  |  |  |
| Positive vs. Negative | 0.57 (0.17-1.94) | | 0.366 | 0.44 (0.12-1.68) | 0.230 |
| Liver metastasis |  |  | |  |  |
| Yes vs. No | 2.27 (1.03-4.99) | | 0.043 | 2.54 (1.01-5.99) | 0.033 |

Table S4. Cox regression analyses between PFS/OS and clinical characteristics of patients with MRG mutant.

Table S4.1. Cox regression analyses of PFS in patients with MRG mutant.

| **Variables** | **Univariate COX** | | **Multivariable COX** | |
| --- | --- | --- | --- | --- |
|  | HR (95% CI) | *P* | HR (95% CI) | *P* |
| HER2 status |  |  |  |  |
| HER2-low vs. HER2-0 | 0.47 (0.23-0.96) | 0.039 | 0.40 (0.17-0.95) | 0.038 |
| Hormone receptor |  |  |  |  |
| Positive vs. Negative | 0.78 (0.19-3.31) | 0.740 | 0.61 (0.13-2.77) | 0.520 |
| Lung metastasis |  |  |  |  |
| Yes vs. No | 1.77 (0.81-3.85) | 0.153 | 1.25 (0.51-3.10) | 0.625 |
| Visceral metastasis |  |  |  |  |
| Yes vs. No | 1.73 (0.84-3.58) | 0.138 | 1.46 (0.61-3.49) | 0.397 |
| Number of metastatic organs |  |  |  |  |
| 2-3 vs. 1 | 2.29 (0.85-6.17) | 0.102 | 3.10 (1.03-9.37) | 0.045 |
| ≥4 vs. 1 | 3.83 (1.19-12.35) | 0.025 | 2.99 (0.85-10.57) | 0.089 |

Table S4.2. Cox regression analyses of OS in patients with MRG mutant.

| **Variables** | **Univariate COX** | | **Multivariable COX** | |
| --- | --- | --- | --- | --- |
|  | HR (95% CI) | *P* | HR (95% CI) | *P* |
| HER2 status |  |  |  |  |
| HER2-low vs. HER2-0 | 0.56 (0.28-1.13) | 0.105 | 0.64 (0.28-1.50) | 0.307 |
| Hormone receptor |  |  |  |  |
| Positive vs. Negative | 0.62 (0.18-2.04) | 0.436 | 0.41 (0.11-1.47) | 0.171 |
| Liver metastasis |  |  |  |  |
| Yes vs. No | 1.70 (0.91-3.19) | 0.099 | 1.59 (0.74-3.40) | 0.236 |
| Radiation therapy |  |  |  |  |
| Yes vs. No | 2.15 (1.12-4.12) | 0.021 | 1.92 (0.90-4.10) | 0.094 |
| Number of metastatic organs |  |  |  |  |
| 2-3 vs. 1 | 2.29 (0.85-6.17) | 0.458 | 1.95 (0.84-4.49) | 0.119 |
| ≥4 vs. 1 | 3.83 (1.19-12.35) | 0.167 | 1.84 (0.67-5.10) | 0.238 |

Table S5. Cox regression analyses between PFS and clinical characteristics of patients with MRG mutant treated with adjuvant endocrine therapy.

| **Variables** | **Univariate COX** | | **Multivariable COX** | |
| --- | --- | --- | --- | --- |
|  | HR (95% CI) | *P* | HR (95% CI) | *P* |
| HER2 status |  |  |  |  |
| HER2-low vs. HER2-0 | 0.35 (0.14-0.86) | 0.023 | 0.35 (0.14-0.88) | 0.026 |
| Radiation therapy |  |  |  |  |
| Yes vs. No | 1.10 (0.53-2.27) | 0.796 | 0.99 (0.48-2.09) | 0.995 |
| Adjuvant chemotherapy |  |  |  |  |
| Yes vs. No | 0.80 (0.32-1.99) | 0.625 | 0.84 (0.33-2.10) | 0.705 |

*PFS progression-free survival, OS overall survival, HR hazard ratio, CI confidence interval,* *MRG metabolic pathway-related gene, mTBI molecular tumor burden index.*
